
JOURNAL INFORMATION
==============================
NLM Title Abbreviation: ScientificWorldJournal
Journal ID: ScientificWorldJournal
NLM Journal ID: 101131163
Journal ID: TSWJ

The Scientific World Journal

ISSN: 2356-6140
EISSN: 1537-744X
Publisher: Wiley

ARTICLE INFORMATION
==============================
PMCID: PMC5917178
DOI: 10.1100/tsw.2006.409
Article ID: 617969
Article version: 1
Subjects: Publisher's Note

Publication of a Special Issue: Superficial Bladder Cancer

Redhead Claire
Publication date: 2006
Electronic publication date: 2006 Oct 23
Volume: 6
First page: 2647
Last page: 2647
Copyright: Copyright © 2006 Claire Redhead.
Copyright year: 2006
License: This is an open access article distributed under the Creative Commons Attribution License, which permits unrestricted use, distribution, and reproduction in any medium, provided the original work is properly cited.
License URL: https://creativecommons.org/licenses/by/3.0/

==============================
Publisher's Note
Superficial Bladder Cancer
*TheScientificWorldJOURNAL* (2006) 6, 2647
TSW Urology
ISSN 1537-744X; DOI 10.1100/tsw.2006.409

TheScientificWorldJOURNAL
www.thescientificworld.com

### Publication of a special issue: Superficial Bladder Cancer

Claire Redhead
*TheScientificWorld UK Ltd*

***E-mail:*** *credhead@thescientificworld.com*

*Published October 23, 2006*

---

TheScientificWorldJOURNAL and TSW Urology are delighted to have published the special issue 'Superficial Bladder Cancer', a collection of peer-reviewed papers covering key topics in this important field. The special issue has been organized by Dr. Ziya Kirkali (Genito-Urinary Group, Dokuz Eylul University, Turkey) and investigates the cancer which is the fourth most common seen in men and which accounts for 10% of all cancers seen in developed countries.

Specific topics covered are:

- Superficial bladder cancer in the prostatic urethra
- Optimal treatment for intermediate and highrisk, non muscle invasive bladder cancer.
- Natural History, Recurrence and Progression in Superficial Bladder Cancer
- The Role of the Surgeon & Trans Urethral Resection in the Treatment of Superficial Bladder Cancer
- New strategies in the treatment of Ta-T1 transitional cell carcinoma (TCC) of the bladder
- Best approach for low risk superficial bladder cancer

The Publisher wishes to express thanks to all of the authors and reviewers, and especially to acknowledge the contribution of Dr. Kirkali in bringing about the publication of these papers.

**This article should be cited as follows:**

Redhead, C. (2006) Publication of special issue. superficial bladder cancer. *TSW Urology* **1(S2),** 45. DOI 10.1100/tswurol.2006.160.

***Corresponding author.**
©2006 with author.
